# Use of Design Thinking to Develop Approaches to Collect and Use Person‐Centered Dementia Care Measures in Low‐Resource Long‐Term Care Settings

**DOI:** 10.1002/alz.084587

**Published:** 2025-01-09

**Authors:** Kirsten Corazzini, Michael Lepore, Nancy Kusmaul, Jing Wang, Alison Rataj, Yoon Chung Kim, Briana Murray, Laura Davie, Eleanor S McConnell, Sarah D Holmes

**Affiliations:** ^1^ University of New Hampshire, Durham, NH USA; ^2^ University of Massachusetts Amherst, Amherst, MA USA; ^3^ University of Maryland Baltimore County, Baltimore, MD USA; ^4^ 4 Library Way, Durham, NH USA; ^5^ University of Maryland Baltimore, Baltimore, MD USA; ^6^ University of Maryland School of Nursing, Baltimore, MD USA; ^7^ Duke University, Durham, NC USA

## Abstract

**Background:**

Low‐resource residential long‐term care (LTC) settings, including settings located in medically underserved and health professional shortage communities, have fewer environmental resources to support high‐quality, robust data collection and use of measures to support person‐centered dementia care (PCC). Further, such settings are more likely to serve older adults from populations that have experienced historic harms related to misuse of personal data, including low‐income and minoritized populations. Design thinking engages community‐members to understand a problem from the end‐user’s perspective (empathize and define), brainstorm new solutions (ideate), and develop proposed solutions (prototype and test). This study describes the development of user‐design workshops to co‐develop, with residents living with dementia (RLWD), their family members and care staff, a person‐centered measurement collection and data sharing infrastructure in low‐resource residential dementia care settings.

**Methods:**

The overall approach is a multi‐method, community‐based, participatory research study with design thinking principles. Stakeholders in each of four low‐resource LTC settings provided in depth perspectives on collecting and sharing data about RLWD to support PCC. Results from qualitative thematic analysis informed the development of user‐design workshops to co‐design feasible and acceptable data collection and use protocols.

**Results:**

Residents, relatives, and staff community members (N = 68 participants) were recruited from 4 low‐resource residential LTC settings (N = 2 rural, 2 urban settings). Journey maps were developed to capture community‐member perspectives by drawing upon themes in each of four key areas: identifying information for care; accessing information for care; sharing information for care; and prioritizing information for care. Journey maps provided the structure to develop co‐design workshop protocols to re‐engage community‐members to empathize, define, ideate, and prototype acceptable and feasible data collection and data sharing protocols of PCC measures.

**Conclusion:**

Findings from this study capture multiple facilitators and barriers to robust data collection and use protocols in resource‐constrained LTC environments. However, findings also demonstrate the potential for user‐centered design approaches to generate novel solutions in ways that honor the perspectives of RLWD, their family members, and the staff who care for them. Ultimately, such approaches contribute to the growth of inclusive research infrastructure to grow capacity for high quality, person‐centered dementia care.

